# Two Coding-Complete Genomes of Tick-Borne Encephalitis Virus Sequenced from *Ixodes persulcatus* Collected in Bulgan, Mongolia

**DOI:** 10.3390/pathogens15040378

**Published:** 2026-04-01

**Authors:** Nora G. Cleary, Nyamdelger Tsevelmaa, Julia E. Paoli, Micah Hoylman, Doniddemberel Altantogtokh, Michael R. Wiley, Jessica D. Wiley, Juan G. Perez Jimenez, Adam Salyer, Irina V. Etobayeva, Nergui Davaasuren, Buyandelger Bolorchimeg, Bandikhuu Amgalanbayar, Carla Mavian, Andrew G. Letizia, Bazartseren Boldbaatar, Michael E. von Fricken

**Affiliations:** 1 Department of Environmental and Global Health, College of Public Health and Health Professions, University of Florida, Gainesville, FL 32610, USA; ncleary@ufl.edu (N.G.C.); paolij20@ufl.edu (J.E.P.); 2 Emerging Pathogens Institute, University of Florida, Gainesville, FL 32610, USA; juanperezjimenez@ufl.edu (J.G.P.J.); cmavian@ufl.edu (C.M.); 3 School of Veterinary Medicine, Mongolian University of Life Sciences, Ulaanbaatar 17029, Mongolia; 1121083@muls.edu.mn (N.T.); altantogtokh@nczd.gov.mn (D.A.); davaasuren.ne@muls.edu.mn (N.D.); 4 Department of Pathology, Microbiology, and Immunology, College of Medicine, University of Nebraska Medical Center, Omaha, NE 68198, USA; mhoylman@unmc.edu (M.H.); mike.wiley@unmc.edu (M.R.W.); 5 National Center for Zoonotic Diseases, Ulaanbaatar 18131, Mongolia; bolorchimeg@nczd.gov.mn (B.B.); amgalanbayar@nczd.gov.mn (B.A.); 6 PathoSeq Bio, Omaha, NE 68101, USA; jess@pathoseqbio.com; 7 Genetics and Genomics Institute, University of Florida, Gainesville, FL 32610, USA; 8 Naval Medical Research Unit INDO PACIFIC (NAMRU-IP), Singapore 759657, Singapore; adam.r.salyer.mil@health.mil (A.S.); irina.v.etobayeva.mil@health.mil (I.V.E.); andrew.g.letizia.mil@health.mil (A.G.L.); 9 Department of Pathology, Immunology and Laboratory Medicine, University of Florida, Gainesville, FL 32610, USA

**Keywords:** tick-borne encephalitis virus, hybrid capture sequencing, Mongolia

## Abstract

Tick-borne encephalitis virus (TBEV) is primarily transmitted by *Ixodes* spp. and poses significant health risks, leading to morbidity and mortality in humans. Two of the five subtypes, Siberian and Far Eastern are known to circulate in Mongolia. In 2021, *Ixodes persulcatus* ticks were collected from Bulgan aimag (province) using flagging and dragging methods and subsequently screened for TBEV using PCR. Positive samples underwent sequencing using an Oxford Nanopore Technologies-based hybrid capture approach, resulting in two coding-complete TBEV genomes from separate tick pools. Phylogenetic analysis classified both genomes within the Siberian subtype, grouping them with other Mongolian sequences from *I. persulcatus* collected in 2014, 2020, 2021, and 2023. The study sequences, PX654173 and PX654174, showed high genetic similarity (99.9% and 99.8%, respectively) to the sequence PQ479142, obtained from *I. persulcatus* ticks in Selenge, Mongolia, in 2021. The estimated time to most recent common ancestor (TMRCA) of the Siberian genotype was approximately 981 CE (95% HPD: 646–1347) with the emergence of a distinct Mongolian clade of TBEV around 1888 CE (95% HPD: 1834–1934). These findings highlight the value of expanded whole-genome sequencing to improve our understanding of TBEV’s genetic diversity and evolutionary history in Central Asia.

## 1. Introduction

Tick-borne encephalitis virus (TBEV), an RNA virus from the *Flavivirus* genus, is prevalent across Eurasia [1,2]. There are approximately 10,000–15,000 TBEV cases reported globally each year, with an average of 20 annual cases in Mongolia, although this number is likely underestimated [3,4]. TBEV clinical manifestations vary widely, ranging from asymptomatic to mild illness to severe meningoencephalitis and death, depending on the subtype [5]. The infection progresses in two phases: the first phase presents with non-specific flu-like symptoms, followed by neurological symptoms in the second phase [6].

The TBEV genome, approximately 11 Kb in size, consists of a single polyprotein that includes three structural proteins (C, prM, and E) and seven non-structural proteins (NS1, NS2A, NS2B, NS3, NS4A, NS4B, and NS5) [7]. The envelope (E) protein is vital for viral cell entry, given that it covers the outside of the viral particle and is the most commonly used target for TBEV subtyping [7,8,9]. Originally, three TBEV subtypes, Far Eastern, Siberian, and Western, were identified, and recently two additional subtypes, Baikalian and Himalayan, have been recognized through phylogenetic analysis [9,10,11]. In Mongolia, located between Russia and China, both the Far Eastern and Siberian subtypes have been detected [4,12]. The Far Eastern subtype is notably more fatal, with the mortality rate ranging from 20 to 40%, compared to 2–3% for the Siberian subtype [5].

TBEV is primarily transmitted by *Ixodes* ticks, with *Ixodes persulcatus* being the main vector in Mongolia [7,13]. Serological studies in humans have detected exposure to TBEV in over half of all Mongolian aimags (provinces) (16 out of 21) and in the capital, Ulaanbaatar. This widespread detection could be explained by transmission across multiple regions, cross-reactivity with other flaviviruses, or the result of a highly mobile population [14,15]. Serological evidence of TBEV exposure was detected in horses, cattle, sheep, and goats in three aimags (Bulgan, Tuv, and Selenge) [16]. While rodents are the main reservoir hosts of TBEV, the presence of antibodies in livestock indicates exposure and suggests that they may contribute to local TBEV dynamics. However, viral RNA has only been detected in *I. persulcatus* ticks in Bulgan and Selenge aimags, indicating a geographically limited range likely tied to vector distribution [4,14,17,18]. In Russia, Siberian TBEV has been found in *I. persulcatus*, *D. nuttalli*, and *D. silvarum*, while Far Eastern TBEV has been isolated from *I. persulcatus* and *Haemaphysalis concinna*, highlighting the potential role of other vector species in transmission; however, this requires additional experimental confirmation [19,20]. TBEV can persist in tick populations through vertical transmission, known as transovarial transmission, from mother to offspring [2]. However, the primary transmission route is through feeding on an infected host or co-feeding with an infected tick on the same host [7].

Genomes of the Siberian subtype have been obtained from *I. persulcatus* ticks collected from 2012 to 2024 in Selenge and Tuv aimags in Mongolia [13,21,22]. Historically, sequencing efforts have focused on the envelope gene for TBEV subtyping, resulting in few complete TBEV sequences from Mongolia [2]. Whole-genome sequences provide a deeper understanding of viral evolution, enable the detection of novel lineages, and improve phylogenetic resolution and epidemiological characteristics beyond the E gene alone [23,24]. Hybrid capture sequencing enables full genome sequencing from samples with low viral loads by using biotinylated oligonucleotide probes to selectively enrich viral nucleic acids prior to sequencing [25]. This method offers advantages over conventional amplicon-based sequencing methods when working with genetically diverse sequences or unknown strains. Although amplicon sequencing provides high sensitivity with on-target primer sequences, it can be vulnerable to amplicon dropout due to primer mismatches caused by viral genetic diversity or primer-dimer formation [26]. Hybrid capture demonstrates greater tolerance to sequence variation, making it particularly effective for RNA virus surveillance, where circulating strains may differ from reference sequences used in primer design. For tick-borne pathogen surveillance, this approach has demonstrated substantial enrichment of pathogen reads and consistent detection across genetically diverse isolates [27]. This study aims to produce coding-complete TBEV genomes from Mongolia using hybrid capture Oxford Nanopore (ONT) sequencing and to apply Bayesian phylodynamic analyses to assess the evolution and spread of the Siberian subtype.

## 2. Materials and Methods

### 2.1. Tick Collection, Homogenization, and Extraction

*Ixodes persulcatus* adult ticks (n = 149) were collected and morphologically identified from a single distinct location in Bulgan aimag in April 2021 via flagging and dragging. Pools of four to five ticks (n = 30) were homogenized and total nucleic acid was extracted using the IndiMag Pathogen Kit (Indical, Leipzig, Germany). PCR was run on the extracted material to screen for TBEV using previously described primers targeting the E gene [22]. Positive PCR pools were converted to cDNA with the AccuPower cDNA synthesis kit following the manufacturer’s instructions for additional testing (Bioneer, Daejeon, Republic of Korea). Tick collection maps were generated using QGIS 3.28.1, and shapefiles were downloaded from the Humanitarian Data Exchange available at https://data.humdata.org/.

### 2.2. Sequencing and Bioinformatics

Samples were processed for sequencing using PathoSeq Bio’s Oxford Nanopore adapted hybrid capture sequencing assay (PathoSeq Bio-ONT) (https://dx.doi.org/10.17504/protocols.io.36wgqd5eovk5/v1 accessed on 16 June 2025), updated for compatibility with R10.4.1 flow cells and LSK114 chemistry (Protocol version 2.7). Briefly, samples were converted to double-stranded cDNA using the NEBNext^®^ RNA First Strand Synthesis Module and NEBNext^®^ Ultra™ II Non-Directional RNA Second Strand Synthesis Module (New England Biolabs, Ipswich, MA, USA). Samples were tagmented using Illumina DNA Prep with Enrichment (S) Tagmentation reagents (small bead-linked transposomes) and amplified using a Nextera-compatible ONT primer mix to add Oxford Nanopore adapter sequences. Libraries underwent enrichment for viral nucleic acid using the Illumina DNA Prep with Enrichment Kit hybridization reagents with probes from the Twist Comprehensive Viral Research Panel (Twist Biosciences, South San Francisco, CA, USA). Enriched libraries were amplified using the TwistDx Amp^®^ Basic Kit (TwistDx, Cambridge, UK) and barcoded with the PCR barcoding expansion kit (PCR Barcoding Expansion Kit, #EXP-PBC096, Oxford Nanopore Technologies Ltd., Oxford, UK) with adapter ligation using ONT Ligation Sequencing kit V14 (Ligation Sequencing kit V14, #SQK-LSK114, ONT, Oxford, UK). Final libraries were pooled and loaded onto an R10.4 minION flow cell (#FLO-MIN114, ONT, Oxford, UK).

Sequencing data was generated and processed on a Dell Alienware laptop using MinKNOW 24.11.10 and in-house tools developed by PathoSeq Bio. Reads were filtered for quality and length, aligned to a curated viral reference database, and grouped by virus. Genome assembly used a combined de novo and reference-guided strategy optimized for ONT reads. Variant calling, consensus generation, and genome masking followed standard long-read analysis procedures. Two coding-complete TBEV genomes were generated. Assembled genomes were submitted to NCBI GenBank under the following accessions: PX654173 (98.88% genome coverage) and PX654174 (97.55% genome coverage). Full details of software, versions, parameters, and database construction are provided in the Supplementary Methods (Methods S1).

### 2.3. Recombination and Phylogenetic Analysis

A reference TBEV sequence dataset for phylogenetic analysis was generated by downloading all publicly available TBEV sequences (n = 485) and their associated metadata from NCBI Virus on 3 August 2025 [28]. The dataset was cleaned by removing sequences shorter than 75% of the full genome length and removing sequences with missing metadata for collection date and location. When TBEV subtype data were unavailable from NCBI Virus, the literature was scanned for subtype metadata. Then, study genomes were aligned with the reference genomes using MAFFT v.7.520, and the alignment was manually polished in AliView v1.28 [29,30]. Recombination analysis was performed with RDP5 using default settings for linear sequences [31]. Recombinant regions were detected and subsequently removed if six of the seven following methods agreed on sufficient evidence of recombination (*p*-value < 0.05): RDP, GENECONV, Chimaera, MaxChi, BootScan, SiScan, and 3Seq [31].

The phylogenetic signal was assessed in IQ-TREE v.2.2.2.7 using likelihood mapping and established guidelines for robust signal (Figure S1). A maximum-likelihood (ML) tree phylogeny was created of the complete genome using IQ-TREE with the Bayesian information criteria (BIC) to infer the best nucleotide substitution model based on the data and 2000 ultrafast bootstrap replicates for support [32,33]. The ML tree was outgroup rooted with the closely related virus Omsk hemorrhagic fever virus (OHFV; NC_005062.1) and visualized in R Studio v4.2.1 using the ggtree v3.2.1 package [34].

### 2.4. Bayesian Phylodynamic Analysis

The reference TBEV dataset of all subtypes was subsampled to only include Siberian subtype sequences, resulting in 105 genomes (Table S1). The temporal signal of the Siberian dataset was assessed using TempEST software v1.5.3 (http://tree.bio.ed.ac.uk/software/tempest/) on ML phylogenies inferred by IQ-TREE using BIC and 2000 ultrafast bootstrap replicates [35]. A Bayesian phylogeny was reconstructed for the Siberian dataset using the Bayesian Evolutionary Analysis Sampling Trees (BEAST) v10.5.0 [36]. Evolutionary rates were estimated with the GTR substitution model, empirical base frequencies, and a gamma site heterogeneity model using BEAUti v10.5.0 version [36]. A constant demographic prior was tested against the Hamiltonian Monte Carlo SkyGrid (HMCS) model and compared a strict molecular clock model to a Hamiltonian Monte Carlo relaxed molecular clock model [37,38]. The Bayes factor was calculated to compare the marginal likelihood of each model using path sampling and stepping-stone methods to determine the best combination of molecular clock and demographic prior (Table S2) [36,39]. Default settings for all other priors and operators were used. Markov chain Monte Carlo (MCMC) samplers were run for 250 million generations with sampling at every 25,000 iterations for three independent runs. Sufficient mixing of the MCMC was assessed by effective sample size (ESS) calculations of ESS > 200 after a 10% burn-in using Tracer v1.7.2. A maximum clade credibility (MCC) tree was obtained to summarize all posterior trees using TreeAnnotator v10.5.0 in BEAST with a 10% burn-in and median node heights. The MCC tree was visualized in R Studio using the ggtree v3.2.1 package [34].

## 3. Results

**Figure 1:**
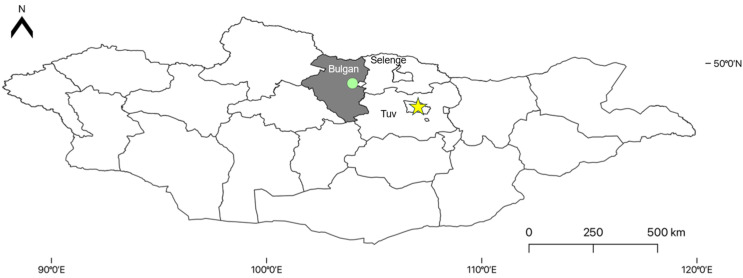
Map of Mongolia showing the location of tick collections at a single location in 2021, represented by a green circle within Bulgan aimag, shaded grey. The yellow star represents the capital city, Ulaanbaatar.

Thirty tick pools were collected in Bulgan aimag, northern Mongolia, in 2021 (Figure 1). RT-PCR screening identified three TBEV-positive pools. Among the three PCR-positive pooled cDNA samples processed for sequencing, two yielded near-complete polyprotein-coding genomes through hybrid capture long-read sequencing. The genomes recovered, designated PX654173 (98.8% genome coverage) and PX654174 (97.6% genome coverage), originated from distinct tick pools. Recombination analysis showed no evidence of recombination in either genome.

**Figure 2:**
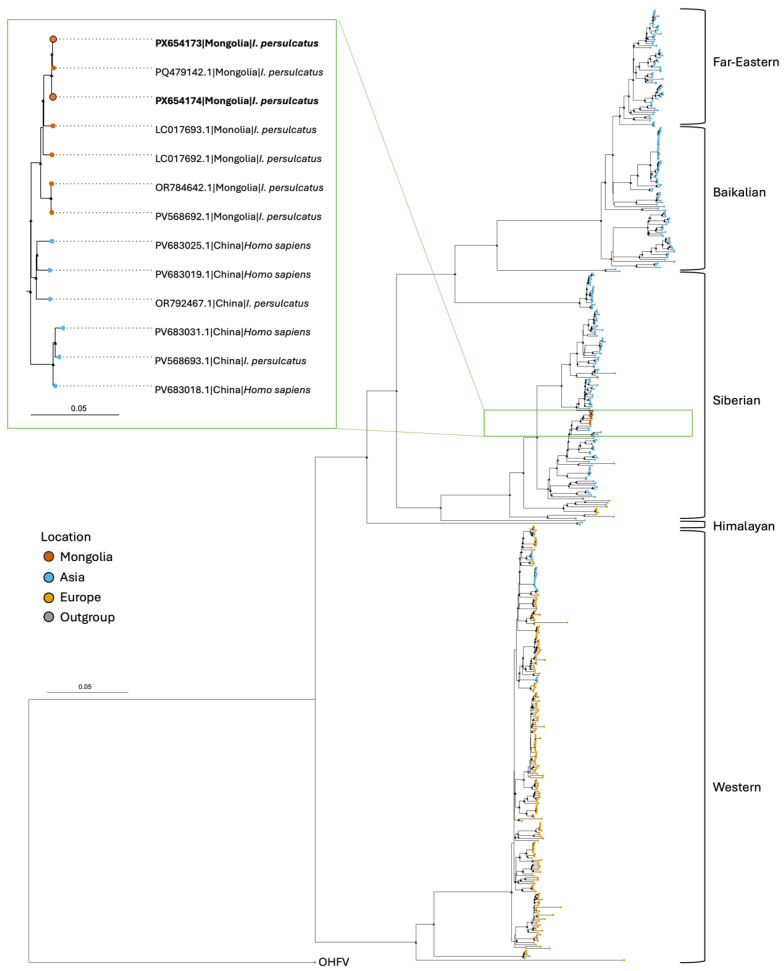
Maximum likelihood phylogeny inferred from alignment of TBEV complete genomes (GTR + F + R4). TBEV subtypes are labeled and tips are colored by geographic location of origin. Black triangles at nodes indicate ultrafast bootstrap values above 90. The subclade containing study sequences PX654173 and PX654174 (bolded) is highlighted in the zoomed box in green. Viruses labeled by GenBank accession number, country of origin, and host. Scale bar in nucleotide substitutions per site. Outgroup rooted by Omsk hemorrhagic fever virus (OHFV; NC_005062.1).

Maximum-likelihood phylogenetic analysis based on complete genome sequences placed both PX654173 and PX654174 genomes within a well-supported monophyletic clade corresponding to the Siberian subtype of TBEV (Figure 2). Within this subtype, the two study genomes clustered most closely with sequences obtained from *I. persulcatus* collected in Mongolia between 2014 and 2023, and China from *I. persulcatus* and humans between 2021 and 2024. PX654173 and PX654174 shared the highest nucleotide identity with PQ479142.1, a sequence obtained from *I. persulcatus* collected in Selenge aimag, Mongolia, in 2020, with 99.9% and 99.8% identities, respectively. Together, these three sequences formed a strongly supported subclade within the Siberian lineage.

**Figure 3:**
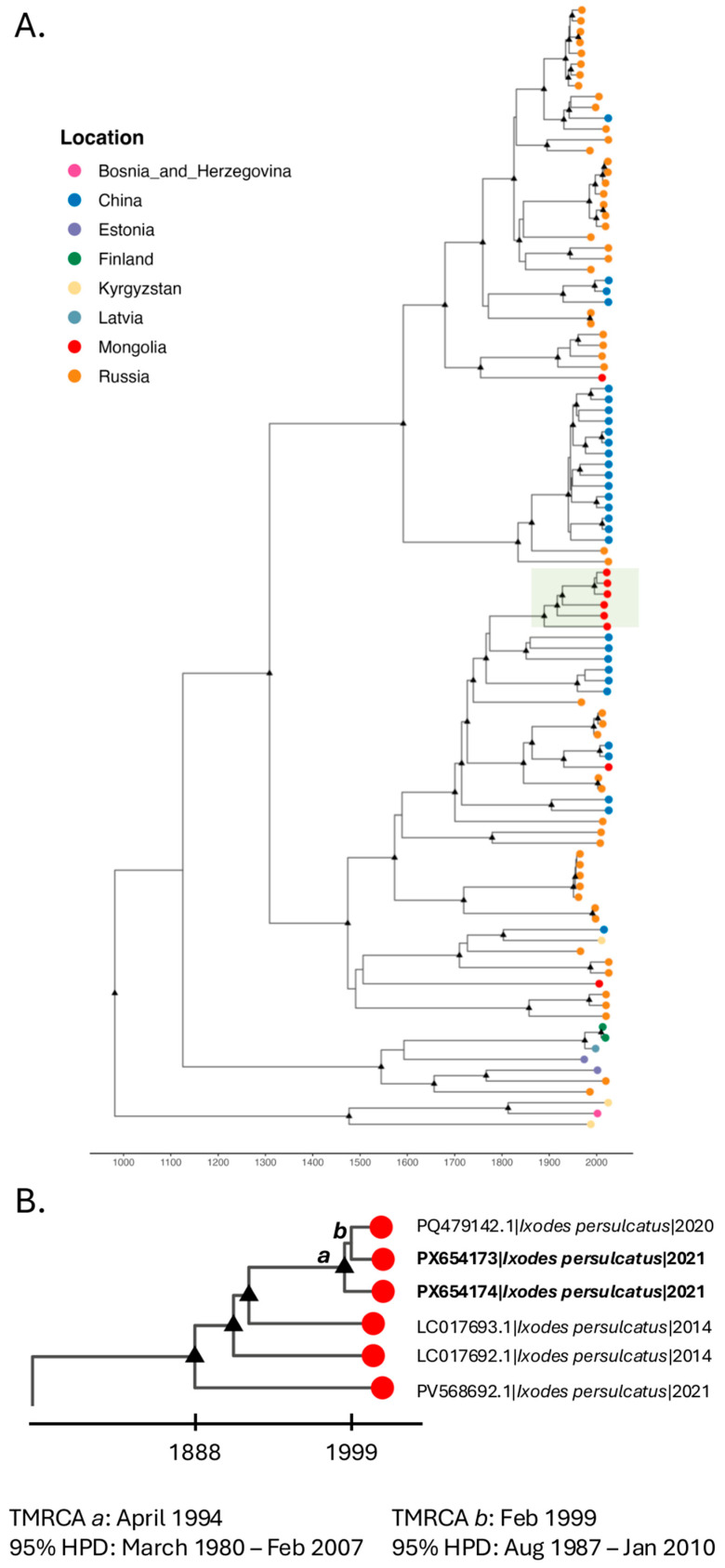
Time-scaled maximum clade credibility (MCC) tree estimated from complete genomes of TBEV Siberian subtype sequences. (**A**) MCC tree inferred using the Hamiltonian Monte Carlo relaxed clock and Hamiltonian Monte Carlo SkyGrid implemented in BEAST X. Tips are colored according to country of origin. Black triangles at internal nodes indicate branches supported by posterior probability > 0.9. The subclade of interest containing sequences from Mongolia generated in this study is highlighted in light green. (**B**) Zoomed view of the highlighted subclade shown in panel A. For selected nodes, a and b, the time to most recent common ancestor (TMRCA), and 95% highest posterior density (HPD) intervals are given. Node a represents the most recent common ancestor of our study sequences and sequence PQ749142.1 collected in Selenge aimag in 2020. Node b represents the common ancestor of study sequence PX654173 and PQ479142.1. Viruses are labelled by GenBank ID, host, and date of collection.

Bayesian phylodynamic analysis of the Siberian subtype dataset estimated the time to the most recent common ancestor (TMRCA) of the Siberian genotype to be approximately 981 Common Era (CE) (95% highest posterior density [HPD]: 646–1347) (Figure 3). Most Mongolian sequences, including PX654173 and PX654174 genomes, clustered within a distinct lineage with an estimated TMRCA of 1888 CE (95% HPD: 1834–1934). Within this lineage, the two study genomes formed a subclade with PQ479142.1, which had an estimated TMRCA of April 1994 (95% HPD: March 1980–February 2007). PX654173 shared a more recent common ancestor with PQ479142 (estimated TMRCA: February 1999; 95% HPD: August 1987–January 2010) than with PX654174. Three sequences collected in Mongolia were found outside of the clade containing our study sequences.

## 4. Discussion

The two coding-complete Siberian TBEV genomes from this study were obtained from *I. persulcatus* ticks from an area in Bulgan aimag with previous TBEV detections. These genomes were most genetically similar to a sequence from eastern Selenge, a neighboring aimag [13]. Given the slow evolutionary rate of TBEV and the short time span between collection periods (2020 and 2021), as well as their close geographic proximity, a high identity between these isolates is expected. Historically, phylogenetic analysis of the Siberian subtype in Mongolia have been limited by a lack of publicly available sequence data; previously published maximum likelihood trees show that Mongolian sequences cluster most closely with sequences from Russia, which borders northern Mongolia [13,40]. In our analysis, we included recently collected Siberian subtype sequences sampled from humans in China in 2024 (PV683025.1, PV683019.1, PV683031.1, PV683018.1) and from *I. persulcatus* in China in 2021 (PV568693.1) and 2023 (OR792467.1). These sequences formed a subclade with the Mongolian sequences, including our study sequences, likely reflecting the availability of additional publicly available TBEV genome data and highlighting the importance of continued genomic surveillance in the region. There have been reports of severe central nervous system disease associated with TBEV infection in local populations in Bulgan [12,17,22]. While this clinical presentation is less commonly associated with the Siberian subtype, infection may increase the risk of chronic disease [41]. Our results are therefore of potential public health importance for understanding TBEV epidemiology in Mongolia. The study sequences from *I. persulcatus* were closely related to both human and tick-derived sequences from China, suggesting a potential for tick-to-human transmission of this Siberian strain. Analyses of TBEV genomes from both ticks and humans provide comprehensive insights into viral diversity and evolution. While the E gene is sufficient for subtyping and often informs disease severity, whole genome analysis can identify novel lineages and mutations that may influence clinical presentation, providing valuable information for clinicians [42,43].

The Siberian subtype has likely circulated in Eurasia for over a thousand years based on the TMRCA of 981 CE. This estimate is slightly older than the 452 CE TMRCA identified in another study of the Siberian subtype of TBEV [44]. This discrepancy may reflect our focus on the Siberian subtype in our BEAST analysis, which excluded other TBEV subtypes. Sustained TBEV circulation in Mongolia is supported by the presence of reservoir hosts, including rodents and competent tick vectors in northern regions [13,22,45]. Between 1834 and 1934, a distinct clade of Siberian TBEV emerged in Mongolia, which includes our study sequences. The high nucleotide similarity and temporal clustering of our study sequences from Bulgan aimag with a sequence collected from *I. persulcatus* in Selenge aimag, Mongolia in 2020 likely reflect the regional circulation of closely related viral strains. This may be facilitated by the dispersal of infected ticks through the movement of large mammals or birds between neighboring aimags. Co-feeding transmission of ticks on vertebrate hosts may also contribute to local viral maintenance. Continued genomic surveillance of TBEV in vector-suitable aimags and in neighboring regions of Russia and China will improve understanding of the evolutionary history of the Siberian subtype in Mongolia.

In northern Mongolia, including the Bulgan aimag, both Siberian and Far-Eastern subtypes of TBEV circulate [46]. Although recombination events between Siberian and Far Eastern strains have been documented in Russia, we found no evidence of recombination in our analyzed sequences [47]. Sustained TBEV transmission in Bulgan is further supported by the presence of multiple tick species capable of harboring TBEV [42]. TBEV has been detected in *I. persulcatus*, *D. nuttalli*, and *D. silvarum* in Mongolia and Bulgan, which contain forest and steppe ecosystems where these tick species thrive [18,47,48,49]. While the role of *Dermacentor* ticks in TBEV transmission remains unclear, cross-species transmission between *D. nuttalli*, *D. silvarum*, and *I. persulcatus* is possible. Experimental studies have shown that *Dermacentor* ticks can harbor and transmit TBEV under experimental conditions [50]. In Tuva, Russia, which borders northwestern Mongolia, the prevalence of *D. nuttalli* and other *Dermacentor* ticks is higher than in *I. persulcatus*, contrary to one Mongolian study where TBEV prevalence in *D. nuttalli* and *D. silvarum* was lower than *I. persulcatus* [51]. Therefore, future TBEV surveillance studies in northwestern Mongolia should also include virome profiling of *Dermacentor* ticks in addition to *Ixodes* ticks [18,52].

The successful recovery of coding-complete TBEV genomes from field-collected tick samples demonstrates the effectiveness of hybrid capture ONT sequencing for tick-borne virus surveillance, even with challenging specimens. These coding-complete genomes enabled comprehensive phylogenetic analysis and accurate placement within the Siberian subtype lineage. This methodological approach is particularly valuable for surveillance programs in regions like Mongolia, where sample collection opportunities are limited and maximizing information recovery from each specimen is critical for understanding viral diversity and evolution. Taken together, this study demonstrates that whole-genome-based TBEV subtyping from tick and human populations is essential for characterizing circulating strains and can inform clinical awareness and public health surveillance.

### Limitations

Only a single distinct location was included in this study over a single time period, limiting the representativeness of TBEV circulating in Mongolia. Two coding-complete genomes were obtained out of three pools that tested positive by RT-PCR, likely due to low viral load in the third sample pool. There is a lack of whole genome sequences of TBEV from Mongolia available in public databases to include in our analyses. In nature, TBEV is maintained and transmitted in a sylvatic system between ticks and small mammals, with humans considered dead-end hosts [53]. Therefore, the effect of this complex system on viral evolution may not be fully captured by BEAST phylodynamic reconstruction, as we did not model the influence of host species, including host jumps and changes to host population size.

## 5. Conclusions

The results of this study support the presence of a distinct lineage of TBEV in Mongolia that likely emerged in the late 19th century. TBEV currently circulating in ticks is similar to previously reported sequences within this clade. This unique Mongolian clade highlights the need for continuous genomic surveillance in border regions to fully understand local TBEV epidemiology. These additional coding-complete genomes of Siberian TBEV from *I. persulcatus* ticks contribute to our understanding of viral evolution in northern Mongolia. Phylogenetic and molecular clock analyses demonstrate that enhanced genomic surveillance can characterize TBEV viral diversity, transmission, and evolution, therefore improving risk assessment and guiding targeted preventative measures. These efforts are critical for safeguarding the health of local populations, travelers, and U.S. and partner military personnel living, visiting, or working in TBEV-endemic areas, including Mongolia and neighboring regions.

## Data Availability

The two sequences are available on GenBank with PX654173 and PX654174 accession numbers.

## Supplementary Materials

The following supporting information can be downloaded at https://www.mdpi.com/article/10.3390/pathogens15040378/s1. Methods S1: Bioinformatic processing; Table S1: Dataset used for BEAST analysis with only the Siberian clade; Table S2: Bayes factor (BF) for selection of molecular clock and demographic prior comparison. Figure S1: Likelihood map used to assess phylogenetic signal of the overall tick-borne encephalitis virus (TBEV) tree.

## Disclaimer

Irina V. Etobayeva (LCDR, MSC, USN), Adam Salyer (LCDR, MSC, USN), and Andrew G. Letizia (CAPT, MC, USN) are military service members and employees of the U.S. Government. This work was prepared as part of their official duties at U.S. Naval Medical Research Unit INDO PACIFIC. The views expressed are those of the author(s) and do not necessarily reflect the official policy or position of the U.S. Army, Department of the Navy, Department of Defense, nor the U.S. Government, nor U.S. Government contractors. Copyright Statement: Title 17 U.S.C. §105 provides that “Copyright protection under this title is not available for any work of the United States Government”. Title 17 U.S.C. §101 defines a U.S. Government work as a work prepared by a military service member or employee of the U.S. Government as part of that person’s official duties.

## Abbreviations

The following abbreviations are used in this manuscript:

BEAST	Bayesian Evolutionary Analysis by Sampling Trees
HPD	Highest Posterior Density
MCC	Maximum Clade Credibility
ONT	Oxford Nanopore Technologies
TBEV	Tick-Borne Encephalitis Virus
TMRCA	Time to Most Recent Common Ancestor
