# Supplementary material for: Two Coding-Complete Genomes of Tick-Borne Encephalitis Virus Sequenced from Ixodes persulcatus Collected in Bulgan, Mongolia
Source: Pathogens. 2026 Apr 1;15(4):378. doi: 10.3390/pathogens15040378 (PMC13119144; doi:10.3390/pathogens15040378)

Supplementary Files

Methods S1. Bioinformatics processing.

Sequencing reads were generated and demultiplexed using MinKNOW (version 25.03.9; Oxford Nanopore Technologies Ltd.) along with adapter and barcode trimming, resulting in FASTQ files for each barcode. Porechop_ABI v.0.5.0 (https://github.com/bonsai-team/Porechop_ABI) was used to remove the Nextera Tagmentation adapter, and reads less than 50 base-pairs in length were removed via chopper v.0.5.0 (https://github.com/wdecoster/chopper). Processed reads were then aligned against a custom database containing curated sequences from NCBI's Virus Database annotated to denote their virus of origin and genome segment (if applicable) using MiniMap2 (v 2.24-r1122).[1] Reads were binned based on the virus classification, and the reference genomes that reads in the bin aligned to were also saved. Bins containing more than 100 reads were assembled. To select a reference genome to assemble reads in each bin to, reads were first assembled using a de novo approach using Miniasm2 and Medaka v.1.7.2 (https://github.com/nanoporetech/medaka), and the contigs generated were aligned back to the group of genomes that the reads in the bin aligned to using MiniMap2.[2] The reference genome with the most contigs aligned to it was selected as the reference, and the contigs were used to correct the genome to make a reference sequence that resembled what was in the sample. If no contigs were generated, the genome with the most individual reads aligned to it was selected as the reference sequence, and then the reference was corrected with the reads in the same way as with contigs. Then, a reference-based assembly approach to the corrected reference genome(s) was used to generate the final assembly. All processed reads for the sample (prior to binning) were realigned to the corrected reference via Minimap2. This step functioned to catch any additional reads that may not have aligned during the initial classification/binning due to divergence to the reference database. Variant calling was performed using Medaka and Longshot v.0.4.5,[3] and variants were filtered to include those found in >50% of the reads at a given position via BCFTools v.1.16 (https://samtools.github.io/bcftools/bcftools.html) and VCFTools v.0.1.16 (https://vcftools.sourceforge.net/). Finally, filtered variants were applied to the consensus genome using BCFTools, and sites with less than 10x reads were masked using SAMTools v.1.16.1 (http://www.htslib.org/) and BEDTools v.2.30.0 (https://bedtools.readthedocs.io/en/latest/).

References

1. Li H. Minimap2: pairwise alignment for nucleotide sequences. *Bioinformatics*. 2018;34(18):3094-3100.

2. Li H. Minimap and miniasm: fast mapping and de novo assembly for noisy long sequences. *Bioinformatics*. 2016;32(14):2103-2110.

3. Edge P, Bansal V. Longshot enables accurate variant calling in diploid genomes from single-molecule long read sequencing. *Nat Commun*. 2019;10(1):4660.

Table S1. Dataset used for BEAST analysis with only the Siberian clade.

GenBank Accession Number	Country	Genotype	Host	Collection Year
MN520113.1	Russia	Siberian	Ochotona alpina	1960
MN520110.1	Russia	Siberian	Microtus maximowiczii	1960
MN520114.1	Russia	Siberian	Pinicola enucleator	1963
MT670183.1	Russia	Siberian	Homo sapiens	1963
JN003206.1	Russia	Siberian	Homo sapiens	1963
MT710347.1	Russia	Siberian	Homo sapiens	1963
MN520111.1	Russia	Siberian	Poecile montanus	1905
MN520112.1	Russia	Siberian	Dendrocopos major	1963
MN115819.1	Russia	Siberian	Homo sapiens	1964
MT710346.1	Russia	Siberian	Homo sapiens	1964
MT710345.1	Russia	Siberian	Homo sapiens	1965
MN115818.1	Russia	Siberian	Homo sapiens	1965
MT670184.1	Russia	Siberian	Homo sapiens	1966
MN115820.1	Russia	Siberian	Homo sapiens	1966
MT710344.1	Russia	Siberian	Homo sapiens	1967
DQ486861.1	Estonia	Siberian	Ixodes persulcatus	1972
KT224352.1	Russia	Siberian	Myodes glareolus	1984
KT224353.1	Russia	Siberian	Ixodes persulcatus	1984
MH645615.1	Russia	Siberian	Ixodes persulcatus	1986
MH645613.1	Russia	Siberian	Ixodes persulcatus	1986
KJ701416.1	Russia	Siberian	Ixodes persulcatus	1986
MH645614.1	Russia	Siberian	Ixodes persulcatus	1986
KJ626343.1	Kyrgyzstan	Siberian	Ixodes persulcatus	1986
JN003207.1	Russia	Siberian	Homo sapiens	1995
GU183382.1	Latvia	Siberian	Mus musculus	1996
KC414090.1	Russia	Siberian	Homo sapiens	1999
LC017691.1	Russia	Siberian	Ixodes persulcatus	1999
KC422663.2	Russia	Siberian	Myodes rutilus	2000
GU183384.1	Estonia	Siberian	Mus musculus	2000

GenBank Accession Number	Country	Genotype	Host	Collection Year
MH645616.1	Bosnia and Herzegovina	Siberian	Ixodes ricinus	2000
JN003208.1	Russia	Siberian	Homo sapiens	2002
MH645612.1	Russia	Siberian	Homo sapiens	2003
MT974474.1	Mongolia	Siberian	Ixodes persulcatus	2004
KM019545.1	Russia	Siberian	Acrocephalus dumetorum	2006
FJ968751.1	Russia	Siberian	Ixodes pavlovskyi	2008
KF826914.1	Russia	Siberian	Ixodes persulcatus	2009
PQ015165.1	Kyrgyzstan	Siberian	Ixodes persulcatus	2009
JN003209.1	Russia	Siberian	Homo sapiens	2010
JQ693478.1	Russia	Siberian	Ixodidae	2010
JQ429588.1	Mongolia	Siberian	Ixodes persulcatus	2010
MG589940.1	Finland	Siberian	Ixodes ricinus	2011
KF823822.1	Russia	Siberian	Ixodes persulcatus	2011
KF826916.1	Russia	Siberian	Culicidae	2011
MH645619.1	Russia	Siberian	Ixodes pavlovskyi	2012
MH645618.1	Russia	Siberian	Ixodes pavlovskyi	2012
KP644245.1	Russia	Siberian	Homo sapiens	2013
OQ565596.1	Russia	Siberian	Homo sapiens	2013
KT321430.1	Russia	Siberian	Homo sapiens	2014
LC017693.1	Mongolia	Siberian	Ixodes persulcatus	2014
MH645617.1	Russia	Siberian	Ixodes persulcatus	2014
LC017692.1	Mongolia	Siberian	Ixodes persulcatus	2014
KP345889.1	China	Siberian	Ixodes scapularis	2014
MF774565.1	Russia	Siberian	Ixodes pavlovskyi	2012
MF043955.1	Russia	Siberian	Homo sapiens	2017
MF043953.1	Russia	Siberian	Homo sapiens	2017
MF043954.1	Russia	Siberian	Homo sapiens	2017
MG589939.1	Finland	Siberian	Homo sapiens	2017
MN114635.1	Russia	Siberian	Homo sapiens	1905
MT344092.1	Russia	Siberian	Ixodes persulcatus	2018
MN114637.1	Russia	Siberian	Ixodes persulcatus	2018
MN114636.1	Russia	Siberian	Ixodes persulcatus	2018
MN542364.1	Russia	Siberian	Ixodes persulcatus	2018
OR827302.1	China	Siberian	Ixodes scapularis	2020
PQ479142.1	Mongolia	Siberian	Ixodes persulcatus	2020
PX654174.1	Mongolia	Siberian	Ixodes persulcatus	2021
PX654174.1	Mongolia	Siberian	Ixodes persulcatus	2021
PV568693.1	China	Siberian	Ixodes persulcatus	2021

GenBank Accession Number	Country	Genotype	Host	Collection Year
PV568692.1	Mongolia	Siberian	Ixodes persulcatus	2021
OP902894.1	Russia	Siberian	Mus musculus	2022
OP902895.1	Russia	Siberian	Mus musculus	1905
PP942933.1	Russia	Siberian	Ixodes persulcatus	2023
PP942934.1	Russia	Siberian	Ixodes persulcatus	2023
PP942931.1	Russia	Siberian	Ixodes persulcatus	2023
PP942932.1	Russia	Siberian	Ixodes persulcatus	2023
PQ553684.1	China	Siberian	Ixodoidea	2023
OR792467.1	China	Siberian	Ixodes scapularis	2023
OR896869.1	Kyrgyzstan	Siberian	Ixodes persulcatus	2023
PQ790051.1	China	Siberian	Homo sapiens	2023
PV683018.1	China	Siberian	Homo sapiens	2024
PV683042.1	China	Siberian	Homo sapiens	2024
PV683034.1	China	Siberian	Homo sapiens	1905
PV683035.1	China	Siberian	Homo sapiens	1905
PV683032.1	China	Siberian	Homo sapiens	2024
PV173737.1	Russia	Siberian	Ixodes persulcatus	2024
PV683038.1	China	Siberian	Homo sapiens	1905
PV683023.1	China	Siberian	Homo sapiens	1905
PV683019.1	China	Siberian	Homo sapiens	2024
PV683024.1	China	Siberian	Homo sapiens	2024
PV683029.1	China	Siberian	Homo sapiens	2024
PV683028.1	China	Siberian	Homo sapiens	2024
PV683036.1	China	Siberian	Homo sapiens	2024
PV683033.1	China	Siberian	Homo sapiens	2024
PV683025.1	China	Siberian	Homo sapiens	2024
PV683039.1	China	Siberian	Homo sapiens	2024
PV683031.1	China	Siberian	Homo sapiens	2024
PV683041.1	China	Siberian	Homo sapiens	1905
PQ014452.1	Russia	Siberian	Ixodes persulcatus	2024
PV788233.1	Mongolia	Siberian	Ixodes persulcatus	2024
PQ790054.1	China	Siberian	Homo sapiens	2024
PQ790055.1	China	Siberian	Homo sapiens	2024
PQ790056.1	China	Siberian	Homo sapiens	2024
PQ790057.1	China	Siberian	Homo sapiens	2024
PV683030.1	China	Siberian	Homo sapiens	2024
PQ790052.1	China	Siberian	Homo sapiens	2024
PQ790053.1	China	Siberian	Homo sapiens	2024

Table S2. Bayes factor (BF) for selection of molecular clock and demographic prior comparison. The log marginal likelihood (log ml) values are shown by Stepping Stone (SS) and Path Sampling (PS) using two clocks: strict clock (SC) or Hamiltonian relaxed clock (HRC) and two demographic priors: constant demographic prior (CONST) or Hamiltonian Monte Carlo SkyGrid demographic prior (HMCSG).

<table>
<tr><th>Model</th><th>log ml SS</th><th>Ln(BF)ss</th><th>log ml PS</th><th>Ln(BF)ps</th></tr>
<tr><td>SC CONST</td><td>-65935.593</td><td rowspan="2">111.474229</td><td>-65935.371</td><td rowspan="2">113.577965</td></tr>
<tr><td>HRC CONST</td><td>-65824.119</td><td>-65821.793</td></tr>
<tr><td>SC HMCSG</td><td>-65916.774</td><td rowspan="2">110.672477</td><td>-65915.367</td><td rowspan="2">110.296492</td></tr>
<tr><td>HRC HMCSG</td><td>-65806.101</td><td>-65805.07</td></tr>
<tr><td>HRC CONST</td><td>-65824.119</td><td rowspan="2">18.0180415</td><td>-65821.793</td><td rowspan="2">16.7232444</td></tr>
<tr><td>HRC HMCSG</td><td>-65806.101</td><td>-65805.07</td></tr>
<tr><td>SC CONST</td><td>-65935.593</td><td rowspan="2">18.8197932</td><td>-65935.371</td><td rowspan="2">20.0047165</td></tr>
<tr><td>SC HMCSG</td><td>-65916.774</td><td>-65915.367</td></tr>
</table>

Figure S1. Likelihood map to assess phylogenetic signal for 485 sequences in the overall tick-borne encephalitis virus (TBEV) tree with alternative topologies (tips), unresolved quarters (center), and partly resolved quartets (edges).

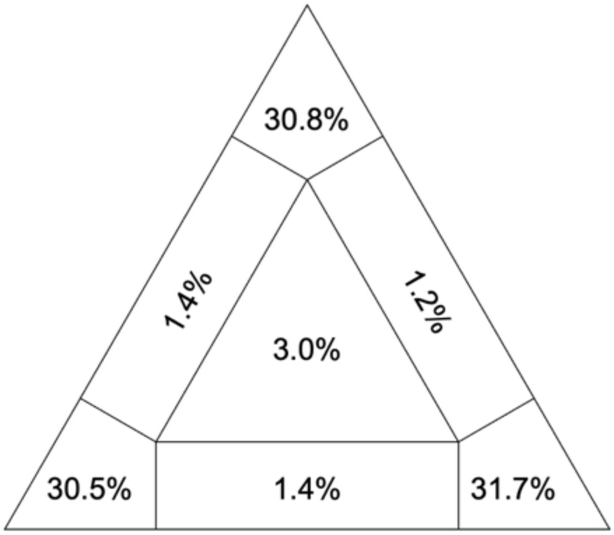

Supplement: Supplementary file 1 [file pathogens-15-00378-s001.zip › pathogens-4211087-supplementary.pdf]
